# Cannula removal with hemostasis secured by thoracoscopic support for accidental central vein puncture: a case report

**DOI:** 10.1093/omcr/omac118

**Published:** 2022-11-24

**Authors:** Satoshi Takamori, Hiroyuki Oizumi, Megumi Nakamura, Jun Suzuki, Akihiro Takeshi, Satoshi Shiono

**Affiliations:** Department of Surgery II, Faculty of Medicine, Yamagata University, Yamagata, Japan; Department of General Thoracic Surgery, Higashiyamato Hospital, Tokyo, Japan; Department of Surgery II, Faculty of Medicine, Yamagata University, Yamagata, Japan; Department of General Thoracic Surgery, Higashiyamato Hospital, Tokyo, Japan; Department of Surgery II, Faculty of Medicine, Yamagata University, Yamagata, Japan; Department of Surgery II, Faculty of Medicine, Yamagata University, Yamagata, Japan; Department of Surgery II, Faculty of Medicine, Yamagata University, Yamagata, Japan; Department of Surgery II, Faculty of Medicine, Yamagata University, Yamagata, Japan

## Abstract

Central venous catheterization is a commonly used procedure for disease management. However, the procedure is not without risks of severe morbidity. We herein report hemostasis for accidental venous puncture using thoracoscopy. A 44-year-old man with short bowel syndrome and chronic renal failure required central venous catheterization for nutritional management and hemodialysis. Right internal jugular vein puncture was performed under ultrasonographic guidance, and the guidewire was inserted into the right atrium under fluoroscopic guidance. However, the operator inadvertently perforated the vein, and the thoracic cavity was entered while inserting the introducer. The patient’s vital signs were stable; therefore, we performed emergency surgery after computed tomography and achieved hemostasis through thoracoscopic surgery. Sufficient caution should be exercised while inserting central venous catheters through a thrombosed internal jugular vein. In some instances of catheter-induced vessel injury, combined surface and thoracoscopic hemostasis may be a reliable and minimally invasive management option.

## INTRODUCTION

Central venous (CV) catheterization is commonly required for patient management. However, therapy can be complicated by morbidities caused by this procedure; these complications can be severe [1–6]. Here, we describe a case of an accidental puncture into the thoracic cavity upon insertion of an introducer into the right internal jugular vein (IJV) and the management strategy used to mitigate the damage.

## CASE REPORT

**Figure 1:**
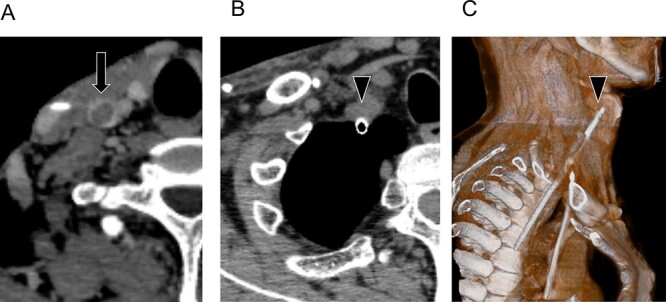
(**A**) Enhanced CT shows the thrombus in the IJV (arrow). (**B**) Intrathoracic perforation of the IJV by the introducer (arrowhead). (**C**) Three-dimensional CT shows the intrathoracic perforation of the introducer (arrowhead).

A 44-year-old man was admitted to our hospital for living-donor kidney transplantation. He had a history of short bowel syndrome due to massive small bowel resection by mesenteric lymphangioma at 3 years of age, and a history of chronic renal disease secondary to renal interstitial disorders associated with short bowel syndrome and chronic dehydration, requiring dialysis since 41 years of age. He received living-donor kidney transplantation; however, he developed acute cellular rejection, and dialysis was required again. Owing to the above condition, the patient has required the installation of multiple venous catheters, with the development of calcifications and thrombi in both brachiocephalic veins and subclavian veins. Computed tomography (CT) revealed thrombosis of the right IJV (Fig. 1A); however, ultrasonography revealed normal blood flow. To establish long-term CV access for dialysis, we planned to insert a Soft Cell™ catheter (Becton, Dickinson and Company, Franklin Lakes, NJ, USA). Under sonographic guidance, the operator attempted catheter insertion into the right IJV. A guidewire was inserted into the superior vena cava (SVC) under fluoroscopy.

During the procedure, the operator noted incorrect orientation of the introducer tip, which appeared positioned within the thoracic cavity. The inner cylinder was withdrawn, revealing no blood flow. A small pneumothorax developed due to air ingress via the outer cylinder, which was immediately capped. The operator suspected inadvertent placement of the introducer into the thoracic cavity; therefore, we were consulted to assist with its removal. The patient’s vital signs were stable, and there was no hemothorax; therefore, immediate CT was performed to localize the perforation. No perforation was found in the right clavicular artery and SVC; however, an intrathoracic perforation through the IJV was observed (Fig. 1B, C). Therefore, we performed a four-port video-assisted thoracoscopic surgery with differential lung ventilation in the left lateral decubitus position under general anesthesia (Video 1). To prevent massive hemorrhage upon removal, we contemplated surface compression and intrathoracic compression in case of a perforation involving only the IJV, and a median sternotomy in case of arterial perforation.

**Figure 2:**
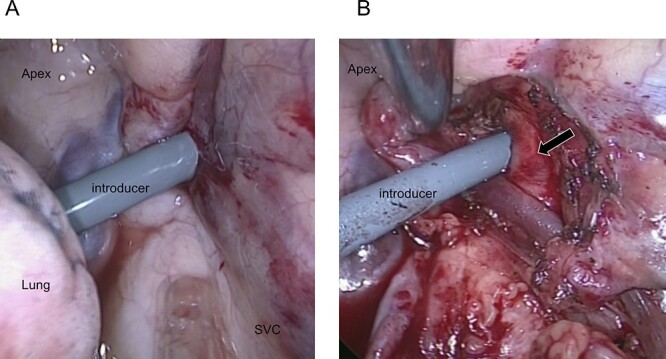
(**A**) Thoracoscopic view of the intrathoracic perforation of the introducer. (**B**) The introducer did not injure the subclavian artery (arrow) and perforated only the IJV.

The introducer did not injure the lung, although an intrapleural hematoma was observed around the perforation site (Fig. 2A). After dissecting the mediastinal pleura, we confirmed a venous perforation without arterial perforation (Fig. 2B).

After removing the introducer, a human fibrinogen-thrombin patch and oxidized regenerated cellulose were applied from within the thoracic cavity to achieve pressure hemostasis. We simultaneously achieved pressure hemostasis from the body surface.

After successful hemostasis, the mediastinal pleura was sutured to coaptation (operative time, 98 min; bleeding, 2 g). The postoperative course was uneventful; 3 days later, a dialysis catheter was inserted through the right femoral vein. During follow-up, 1.5 years after the surgery, the patient was alive.

## DISCUSSION

A CV catheter can be lifesaving; however, its insertion is associated with potentially severe complications, including hemo- and/or pneumothorax, nerve injury, major bleeding, catheter-related bloodstream infection and venous thrombosis [1–6]. Patients requiring CV catheters often have complex pathologies, and the morbidities associated with CV insertion further complicate treatment. Prospective randomized trials and meta-analyses have suggested that the use of ultrasonography when placing IJV catheters reduces complication rates [4–6]. Our operator performed venipuncture using ultrasonography; however, intrathoracic perforation occurred.

In this case, CT showed an IJV thrombus related to a prior CV catheterization; however, ultrasonography confirmed blood flow in the IJV, suggesting that the guidewire could be placed into the right atrium under fluoroscopic guidance. When inserting the introducer, an incorrect insertion angle might have led to contralateral perforation, and thrombus may have contributed. Applying excessive force when the introducer cannot be advanced is dangerous. Care is required during catheter insertion into a thrombosed vessel, and puncture should be avoided. In this case, inserting a temporary femoral venous dialysis catheter may have been considered. In addition, percutaneous transluminal angioplasty with or without stent placement has been suggested as the preferred approach to CV stenosis.

If a large venous catheter perforates into the intrathoracic cavity, a massive hemopneumothorax may occur, requiring surgical repair [7–9]. In this case, the patient’s vital signs were stable, and no hemothorax was observed on fluoroscopy. Therefore, we performed a CT scan to examine the perforation in detail. If the catheter is removed immediately after vessel perforation, massive bleeding may result, and the injured vessel may be difficult to identify.

With massive venous bleeding, or subclavian artery perforation, thoracoscopic hemostasis may be dangerous. These injuries are conventionally managed using open surgical exploration [7, 8]. However, reports have demonstrated the effectiveness of thoracoscopic surgery for catheter-related subclavian artery or vein injury [9, 10]. In this case, the patient’s vital signs were stable after the inadvertent puncture. After thin-slice (0.5 mm) CT examination of the traumatized area, we suspected an IJV puncture without hemothorax. Even if only the IJV is injured, if a large catheter reaches the thoracic cavity, the negative pressure in the thoracic cavity may cause massive hemothorax. In addition, confirming that only the IJV is injured is impossible if enhanced CT is foregone because of the emergency. To date, reports of thoracoscopic hemostasis have identified hemothorax after CV catheterization [9, 10]. In this case, we suspected a misplacement into the thoracic cavity during the insertion of the introducer. In such cases, if the introducer is removed immediately, massive bleeding may occur, and it may be difficult to identify the injured vessel. Therefore, the catheter was removed with thoracoscopic support in anticipation of massive bleeding due to subclavian artery or vein injury.

In conclusion, caution should be exercised while inserting CV catheters through a thrombosed IJV. Even with correct guidewire placement under fluoroscopy, the introducer might be inserted incorrectly. In catheter-related vessel injury, combined external and thoracoscopic hemostasis may be a reliable and minimally invasive option.

## AUTHOR CONTRIBUTIONS

ST: Conceptualization; Data curation; Writing—original draft. HO: Conceptualization; Project administration; Supervision; Writing—review & editing. JS: Supervision; Writing—review & editing. MN: Supervision; Writing—review & editing. AT: Supervision; Writing—review & editing. SS: Supervision; Writing—review & editing. All authors read and approved the final manuscript.

**LIST OF ABBREVIATIONS**
CT: Computed tomography
  CV
  Central venous
  IJV
  Internal jugular vein
  SVC
  Superior vena cava

## Supplementary Material

video_1_omac118

## Data Availability

Not applicable.
